# Titanium Surface Roughness Mediated Macrophages Polarization-Influenced Osteogenic Differentiation of Periodontal Ligament-Derived Mesenchymal Stromal Cells

**DOI:** 10.1055/s-0045-1804889

**Published:** 2025-05-07

**Authors:** Dodi V. Tambun, Jovanka Tanandika, Carlita Carlita, Fakhrana A. Ayub, Ratna Ramadhani, Ratna Sari Dewi, Ariadna Djais, Ferry Gultom, Sunarso Sunarso, Lisa R. Amir

**Affiliations:** 1 Dentistry Study Program, Faculty of Dentistry, Universitas Indonesia, Jakarta, Indonesia; 2 Department of Prosthodontics, Faculty of Dentistry, Universitas Indonesia, Jakarta, Indonesia; 3 Department of Oral Biology, Faculty of Dentistry, Universitas Indonesia, Jakarta, Indonesia; 4 Department of Dental Material, Faculty of Dentistry, Universitas Indonesia, Jakarta, Indonesia

**Keywords:** titanium surface roughness, macrophages polarization, PDL MSCs, osteogenesis

## Abstract

**Objectives:**

Implant surface topography significantly influences cell behavior, including macrophages and bone cell interactions. The polarization of macrophages, key immune cells, is influenced by implant surface characteristics. This research aimed to examine periodontal ligament mesenchymal stromal cells (PDL MSCs) responses to the polarized macrophages induced by titanium surface roughness.

**Materials and Methods:**

RAW 264.7 macrophages were cultured with various surface roughness of titanium disks. Macrophage adhesion and polarization were evaluated by scanning electron microscope, gene expressions profiling, and flow cytometry. PDL MSCs were treated with conditioned medium of macrophages and analyzed with 3-[4,5-dimethylthiazol-2yl]-2,5-diphenyl-2H-tetrazolium bromide assay, real-time polymerase chain reaction, and Alizarin red staining.

**Statistical Analysis:**

Data was statistically analyzed using GraphPad Prism 9 for Windows 11. The one-way analysis of variance test was used to compare the groups. Dunn post hoc test was used to compare the difference between the groups when appropriate. Significance was accepted when *p* < 0.05.

**Results:**

Medium surface roughness (Ti-MR) consistently inhibited tumor necrosis factor-α, interleukin-1β (IL-1β), and IL-6 gene expressions ( *p* < 0.001) and upregulated transforming growth factor-β, vascular epithelial growth factor, and IL-10 expressions ( *p* < 0.01). Confirmatory flow cytometry analysis showed consistent results, with Ti-HR and Ti-MR exhibiting the highest population of CD163+ cells (99.1 and 90.7%, respectively), while Ti-LR exhibited the lowest M1/M2 ratio (0.93). Furthermore, treatment of RAW 264.7 conditioned medium increased osteopontin, alkaline phosphatase, collagen type-1 A-1 chain, osteocalcin, runt-related transcription factor-2, and bone sialoprotein gene expressions and calcium deposition ( *p* < 0.01).

**Conclusion:**

Titanium implant surface topography influences macrophage polarization and osteogenic differentiation of PDL MSCs, with Ti-MR being the most effective in polarizing macrophages toward M2 and inducing optimal osteogenic responses from PDL MSCs.

## Introduction

The surface topography of titanium implants plays a critical role in influencing cell behavior, including the responses of bone cells and macrophages. Various studies have demonstrated that specific surface features, such as roughness and texture, can modulate cell adhesion, proliferation, and differentiation, which are crucial for successful osseointegration. 1 2 3

Macrophages, key players in the immune response, exhibit a range of polarization states that significantly impact tissue healing and regeneration. Macrophages can adopt different activation states or phenotypes, broadly classified into proinflammatory M1 and anti-inflammatory M2 phenotypes 4 and can be influenced by the physical characteristics of the implant surface. 5 6 M1 macrophages are generally proinflammatory, producing cytokines like tumor necrosis factor-α (TNF-α), while M2 macrophages are associated with anti-inflammatory responses and tissue repair, secreting factors such as interleukin (IL)-10 and Arg-1. 7 8 The ability of implant surfaces to sway macrophage polarization thus holds significant implications for bone healing and integration of implants. 9 10

Periodontal ligament mesenchymal stromal cells (PDL MSCs) are a population of stromal cells capable of differentiating into various cell types involved in periodontal tissue regeneration. These cells have been shown to interact closely with macrophages, a type of immune cell crucial for both inflammatory responses and tissue repair processes. The interaction between PDL MSCs and macrophages is vital for regulating the immune microenvironment in periodontal tissues.

Macrophage polarization has a profound impact on the osteogenic differentiation of PDL MSCs. M1 macrophages, which produce proinflammatory cytokines such as TNF-α and IL-1β, can create an inflammatory microenvironment that inhibits MSCs proliferation and osteogenesis. 11 12 In contrast, M2 macrophages secrete anti-inflammatory cytokines like IL-10 and transforming growth factor-β (TGF-β), promoting a regenerative environment that enhances MSCs differentiation into osteoblasts. 13 14 PDL MSCs exposed to M2 macrophage-conditioned media exhibit an increase in mineralization and higher expression of osteogenic markers, underscoring the importance of M2 macrophages in facilitating bone regeneration. 15 16

Despite the established importance of surface topography on cell behavior and macrophage polarization, the specific effects of titanium surface roughness on PDL MSCs-mediated macrophage polarization and their subsequent roles in osteogenesis remain unclear. Our study aims to bridge this gap by examining whether polarized macrophage induced by surface topographical cues of titanium implants influences the osteogenic potential of PDL MSCs. Understanding these interactions is crucial for optimizing implant design to enhance bone regeneration and ensure long-term success of dental implants.

## Materials and Methods

### Preparation and Surface Characterization of Titanium Disks

**Fig. 1:**
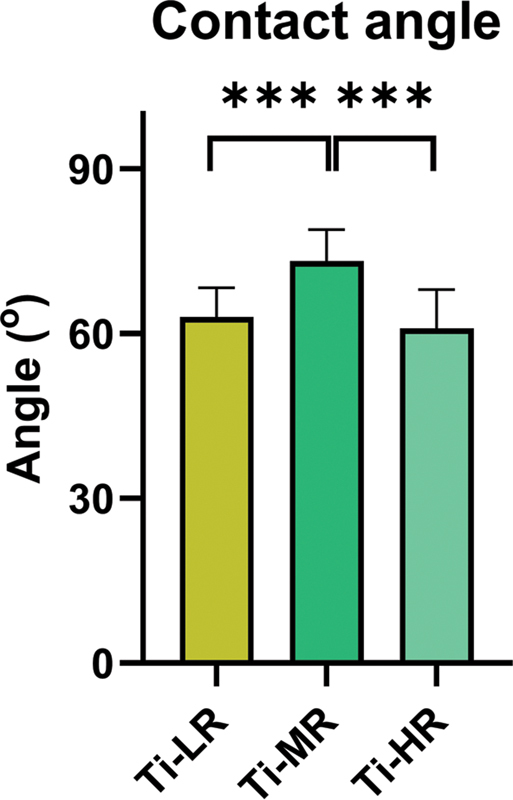
The wettability of surfaces was tested by water contact angle using low bond-axisymmetric drop shape analysis (LB-ADSA) method ( *n* = 6). A significant difference between groups was indicated by *** *p* < 0.001.

Titanium disks (diameter 12 mm; thickness 2 mm) were obtained from Pudak Scientific, Indonesia, with various surface roughness. Scanning electron microscope (SEM; FEI-quanta 650, Zeiss, Germany) analysis revealed roughness values (Ra) of low roughness (Ti-LR), medium roughness (Ti-MR), and high roughness (Ti-HR) titanium were 0.82, 2.42, and 3.66 μm, respectively. The contact angles measured using low bond-axisymmetric drop shape analysis with ImageJ (National Institutes of Health, United States), ranged from 59 to 73 degrees, indicating a moderate hydrophilicity ( Fig. 1 ). All disks were sterilized by 25 kGy gamma irradiation prior to cell seeding.

### RAW 264.7 Cell Culture

RAW 264.7 cells were cultured in Iscove's Modified Dulbecco's Medium supplemented with 10% heat-inactivated fetal bovine serum (FBS), 1% penicillin-streptomycin, and 1% amphotericin-B and incubated at 37°C and 5% CO _2_ in 95% humidified atmosphere. All medium and reagents in this study were obtained from Gibco, United States, unless otherwise specified. Titanium Ti-LR, Ti-MR, and Ti-HR were placed in 24-well plates. A total of 1 × 10 ^5^ RAW 264.7 cells suspended in 50 μL were added to titanium disks in each well ( *n* = 3), ensuring complete coverage of the titanium by the cells, and incubated for 1 hour for cell adhesion and stabilization. Cells cultured in medium only served as the control group. Independent experiments were repeated at least two times and performed in triplicate.

### Cell Adhesion Morphology

Following cells cultured with titanium disks for 24 hours, cells were fixed with 2.5% glutaraldehyde for 2 hours and dehydrated in a gradient series of ethanol. Cell adhesion on the different samples was examined using SEM and immunofluorescence staining. Samples were stained with Phalloidin blue (Abcam) at 1:1,000 dilution for 90 minutes. Nuclear staining was carried out using 5 μg/mL Hoechst 33342 (Abcam), incubated for 60 minutes, mounted with mounting medium, and observed using an inverted fluorescence microscope (Zeiss AxioCam MRc5, Germany).

### Flow Cytometry

The surface markers of M1 macrophages (CD86) and M2 macrophages (CD163) were examined using flow cytometry. Briefly, RAW 264.7 macrophages cultured on different samples were isolated by trypsinization and washed with phosphate-buffered saline (PBS) two times. Subsequently, the cells incubated with allophycocyanin-conjugated CD163 (Elabscience) and phycoerythrin-conjugated CD86 (Elabscience) for 15 minutes. Last, cells were washed with PBS and then analyzed using a flow cytometer.

### Real-Time Polymerase Chain Reaction

Real-time polymerase chain reaction (RT-PCR) was performed using primers as listed in Table 1 . The cells were lysed with Trizol (Thermo Fisher, United States) at 4°C overnight. Complementary deoxyribonucleic acid (cDNA) was synthesized from total ribonucleic acid (RNA) and standardized to concentration 7.5 ng/μL in thermal cycler (Thermo Fisher). RT-PCR was conducted using 5 μL of sample cDNA with a denaturation step at 85°C for 15 seconds, annealing and extension at 60°C for 1 minute, for a total of 45 cycles. Target gene expression was normalized to the housekeeping gene glyceraldehyde 6-phosphate (GAPDH) and 18s ribosomal RNA (18s), and data were calculated using the 2 ^‒ΔΔCt^ method.

**Table 1:** Primer sequences used for RT-PCR

Target genes	Forward primer	Reverse primer
IL-1B	5′-AGCCCATCCTCTGTGACTCAT-3′	5′-CATTGAGGTGGAGAGCTTTC-3′
IL-6	5′-CTTGGGACTGATGCTGGTG-3′	5′-TTCCACGATTTCCCAGAGA-3′
TNF-a	5′-TCGTAGCAAACCACCAAGTG-3′	5′-CCTTGAAGAGAACCTGGGAGT-3′
IL-10	5′-GAGAAGCATGGCCCAGAAATC-3′	5-GAGAAATCGATGACAGCGCC-3′
TGF-B	5′-TGGAGCAACATGTGGAACTC-3′	5′-TGCCGTACAACTCCAGTGAC-3′
VEGF	5′-TTACTGCTGTACCTCCACC-3′	5′-ACAGGACGGCTTGAAGATG-3′
GAPDH	5′-TGTGTCCGTCGTGGATCTGA-3′	5′-CCTGCTTCACCACCTTCTTGAT-3′
ALP	5′- GCAACTTCCAGACCATTGGC -3′	5′-TCCCACTGACTTCCCTGCTT-3′
OPN	5′-ACATCCAGTACCCTGATGCTACAG-3′	5′-TGGCCTTGTATGCACCATTC-3′
Col1A1	5′- TCTGCGACAACGGCAAGGTG -3′	5′-GACGCCGGTGGTTTCTTGGT-3′
OCN	5′-GCTACCTGTATCAATGGCTG-3′	5′-GGAAGAGGAAAGAAGGGTG-3′
BSP	5′-GATTTCCAGTTCAGGGCAGTAG-3′	5′-CCCAGTGTTGTAGCAGAAAGTG-3′
Runx2	5′-ATGCTTCATTCGCCTCAC-3′	5′-ACTGCTTGCAGCCTTAAAT-3′
18s	5′- TGGACAACAAGCTCCGTGAA -3′	5′-TCGAGGTAGGCAGGGAGATG-3′

Abbreviations: 18s, 18s ribosomal RNA; ALP, alkaline phosphatase; BSP, bone sialoprotein; Col1A1, collagen type-1 A-1 chain; GAPDH, glyceraldehyde 6-phosphate; IL-10, interleukin-10; IL-1β, interleukin-1β; IL-6, interleukin-6; OCN, osteocalcin; OPN, osteopontin; RT-PCR, real-time polymerase chain reaction; Runx2, runt-related transcription factor 2; TGF-β, transforming growth factor-β; TNF-α, tumor necrosis factor-α; VEGF, vascular epithelial growth factor.

### PDL MSCs Regulation Toward RAW 264.7 Response

The conditioned medium (CM) of RAW 264.7 macrophage cultured with titanium disks for 24 hours were collected. PDL MSCs under the sixth passage were cultured in Alpha Modified Eagle's Medium supplemented with 10% FBS, 1% penicillin-streptomycin, and 1% amphotericin-B. PDL MSCs were seeded at a density of 1 × 10 ^4^ cell per well in a 96-well plate and subjected to serum starvation for 24 hours. Macrophages CM were added in medium at 1:1, 1:3, and 3:1 ratio for 24 hours. A proliferation assay using 5 mg/mL of 3-[4,5-dimethylthiazol-2yl]-2,5-diphenyl-2H-tetrazolium bromide (MTT; Sigma-Aldrich, United States) was performed to evaluate cell proliferation. The absorbance values were determined at 600 nm.

Alizarin red staining and RT-PCR were performed to evaluate differentiation. PDL MSCs were cultured in medium with macrophage CM at 1:1 ratio for 72 hours, followed by cultured in an osteogenic medium containing 10 mM β-glycerophosphate, 100 nM dexamethasone, and 0.2 mM L-ascorbic acid for 7 and 14 days. Cells were fixed with 4% paraformaldehyde and stained with 2% Alizarin red (Thermo Fisher) solution. Images of the stained calcium nodules were captured using a digital camera and light microscope (Zeiss, Germany). The mineralized nodules were dissolved in 10% hexadecylpyridinium chloride (Thermo Fisher) and quantified using a microplate reader at 600 nm.

### Statistical Analysis

Data was statistically analyzed using GraphPad Prism 9 for Windows 11. The one-way analysis of variance test was used to compare the groups. Dunn post hoc test was used to compare the difference between the groups when appropriate. Significance was accepted when *p* < 0.05.

## Results

### RAW 264.7 Macrophage Adhesion

**Fig. 2:**
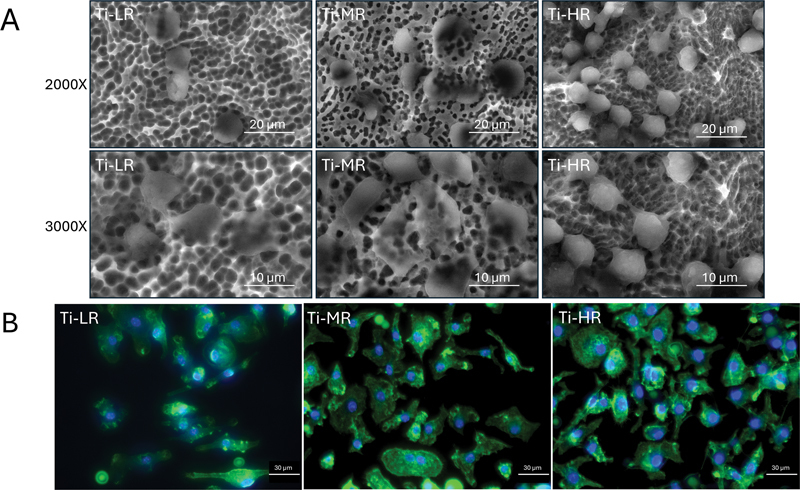
Cell morphology. ( **A** ) Representative image of RAW 264.7 cell morphology on titanium surfaces was observed using scanning electron microscope (SEM) at 2,000× and 3,000× magnification (the scale bar is 20 and 10 μm, respectively). ( **B** ) Immunofluorescence microscope observation 40× magnification (the scale bar is 30 μm).

RAW 264.7 macrophages were cultured on various titanium disks and observed using SEM and immunofluorescence ( Fig. 2 ). The cells cultured on Ti-LR displayed a morphology that was quite similar to those cultured on Ti-HR, characterized by a rounded shape with numerous filopodia projections. Despite this similarity in cell shape, it was noted that the cell colonies formed on the Ti-HR substrate were more abundant compared to those on Ti-LR. The macrophages on Ti-MR appeared more flattened. Instead of typical filopodia, the cell attachment on Ti-MR was characterized by lamellipodia, evidenced by broad, sheet-like extensions, at the leading edge of the cells ( Fig. 2A ). The fluorescence microscopy findings further confirmed cell morphologies that were observed under SEM. Lamellipodia in cells cultured on Ti-MR were clearly depicted by broad sheet-like extensions. In addition to Ti-MR, flattened cells were also observed in some cells on Ti-LR and Ti-HR. Cells on Ti-LR exhibited more diverse morphologies, including elongated cells and some that were flattened. The majority of cells on Ti-HR showed less pronounced flattening compared to those on Ti-MR, with cell adhesion on Ti-MR being evident through thin actin filaments extending from the cell edges to form pseudopodia ( Fig. 2B ).

### Titanium Surfaces Induce Specific Macrophage Polarization

**Fig. 3:**
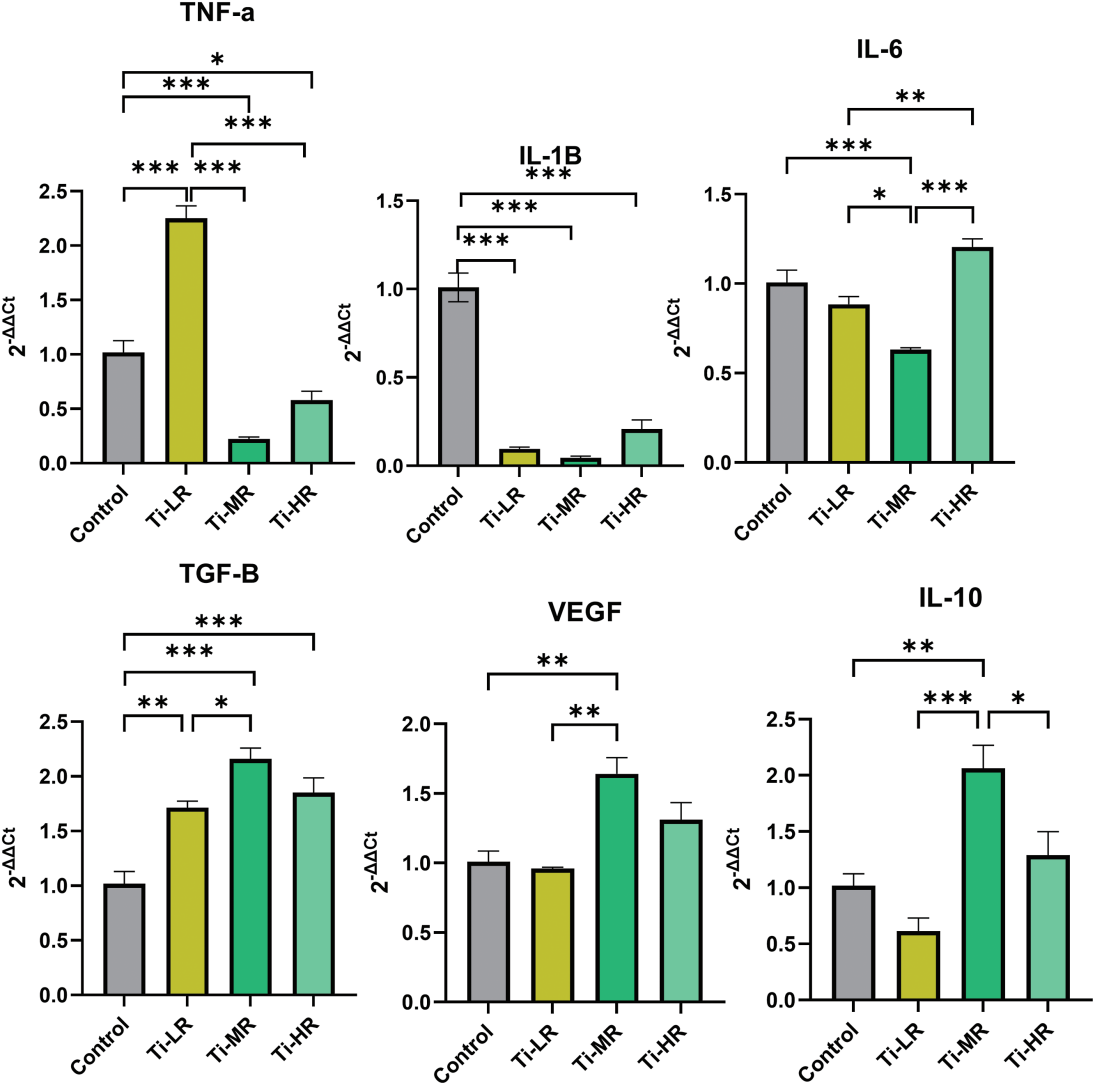
M1/M2 macrophage gene expression of RAW 264.7 cells ( *n* = 3). A significant difference between groups was indicated by * *p* < 0.05, ** *p* < 0.01, and *** *p* < 0.001.

The gene expression of M1 macrophage (IL-1β, IL-6, and TNF-α) and M2 macrophage markers (IL-10, vascular epithelial growth factors [VEGFs], and TGF-β) was further investigated to analyze macrophage polarization on each titanium surface ( Fig. 3 ). The Ti-LR surface exhibited a significant increase in TNF-α expression but decrease in IL-1β expression. Ti-MR and Ti-HR showed a significant decrease in IL-1β and TNF-α expressions. In contrast, the analysis of M2 markers revealed distinct patterns. There was a significant increase in the expression of VEGF, IL-10, and TGF-β genes on the Ti-MR surface. All titanium groups show an increase in TGF-β expression.

**Fig. 4:**
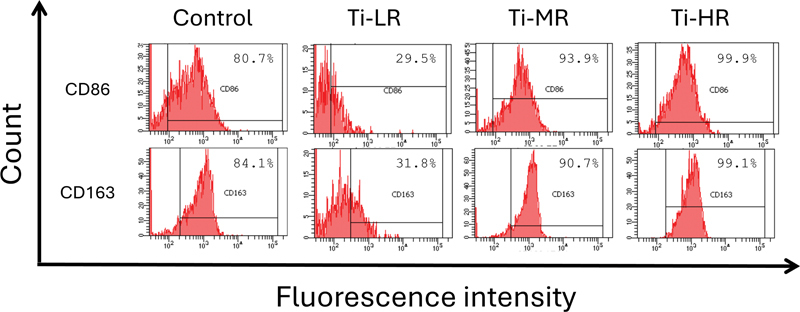
Polarization of macrophages was evaluated by the expressions of M1 (CD86) and M2 (CD163) markers using flow cytometry.

To further confirm the phenotype of polarized macrophages on different samples, CD86 was selected as markers for M1 macrophages, while CD163 was used as markers for M2 macrophages. Flow cytometry analysis was conducted to evaluate macrophage polarization. As shown in Fig. 4 , the proportion of cell populations varied across samples compared to control group. Ti-LR exhibited a reduction in the proportion of CD86 ^+^ and enhancement of CD163 ^+^ . In contrast, Ti-MR and Ti-HR displayed an increase in the proportion of all cell populations. Further analysis was conducted by comparing the M1/M2 ratio across all groups. The results revealed the following trend: Ti-LR (0.93) < control (0.96) < Ti-HR (1.00) < Ti-MR (1.03).

### Influence of RAW 264.7 Macrophages on the PDL MSCs Proliferation and Osteogenic Differentiation

**Fig. 5:**
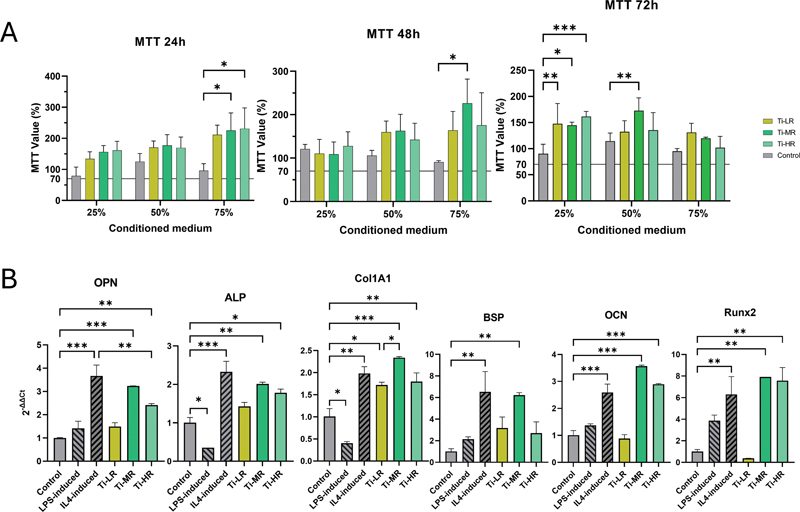
Comparison of cell proliferation and osteogenesis of periodontal ligament mesenchymal stromal cells (PDL MSCs). ( **A** ) Comparison of cell proliferation with 3-[4,5-dimethylthiazol-2yl]-2,5-diphenyl-2H-tetrazolium bromide (MTT) value of 70% set as threshold for cytotoxicity ( *n* = 3). ( **B** ) Osteogenesis-related gene expression of PDL MSCs ( *n* = 3). A significant difference between groups was indicated by * *p* < 0.05, ** *p* < 0.01, and *** *p* < 0.001.

**Fig. 6:**
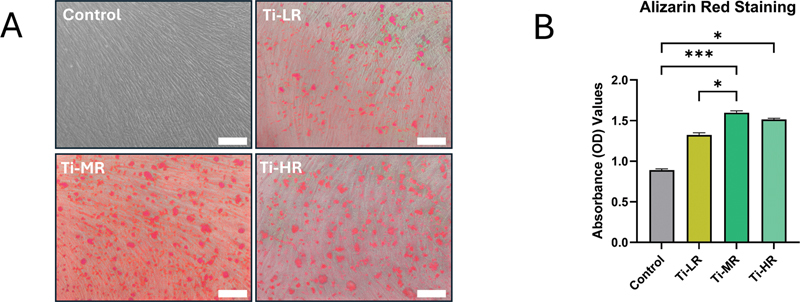
Representative images of Alizarin red staining ( **A** ; the scale bar is 200 μm) and ( **B** ) quantitative analysis of calcium deposition ( *n* = 3). A significant difference between groups was indicated by * *p* < 0.05, ** *p* < 0.01, and *** *p* < 0.001.

The result of MTT assay and osteogenic gene expressions are presented in Fig. 5 . All groups demonstrated MTT values above 70% indicating that the titanium samples are biocompatible ( Fig. 5A ). RAW 264.7 macrophage CM significantly increased osteogenic gene expression in the Ti-MR and Ti-HR groups compared to the control ( Fig. 5B ). Interestingly, PDL MSCs exposed to macrophage CM derived from RAW 264.7 cells stimulated with IL-4 exhibited elevated expression of all three M2 markers, and this increase was not significantly different from that observed in the Ti-MR group. Furthermore, an increase in calcium deposition was demonstrated in the Ti-MR and Ti-HR groups ( Fig. 6A and B ).

## Discussion

The present study evaluated the impact of polarized macrophage induced by surface topographical cues of titanium implants on the osteogenic potential of PDL MSCs. The characterization of titanium surfaces reveals intriguing insights into the complex relationship between surface roughness, wettability, and cellular behavior. Contrary to the traditional view that increased hydrophilicity enhances cell adhesion and proliferation, 17 Ti-MR demonstrated superior cell performance. Intermediate roughness levels, like those found in Ti-MR, provide a balanced surface topology that facilitates effective mechanical interlocking and supports better cell attachment and proliferation. 18 19 The Ti-MR's roughness creates an optimal microenvironment, promoting favorable interactions between cells and the substrate. 20

The morphology of RAW 264.7 macrophages were varying in the different titanium surfaces, highlighting the impact of surface characteristics on cell behavior. Our results showed that macrophages on both Ti-LR and Ti-HR surfaces generally exhibited a rounded morphology with numerous filopodia projections. Despite similar morphology, macrophages formed more abundant colonies on Ti-HR compared to Ti-LR. This suggests that while surface roughness does not significantly alter the initial shape of macrophages, it influences cell proliferation and colony formation. 6 7 In contrast, macrophages on Ti-MR surface displayed a markedly different, flattened morphology characterized by lamellipodia rather than filopodia. These broad, sheet-like extensions suggest a different mode of attachment and interaction with the substrate, potentially indicating a distinct activation state or functional response. 21 Such morphological changes might be associated with macrophage polarization states, where the presence of lamellipodia could signify a transition to an anti-inflammatory M2 phenotype, which is typically involved in tissue repair and remodeling. 22 This is consistent with previous studies that have demonstrated that surface roughness and topography can influence macrophage polarization and function. 23

Consistent with the gene expression results, flow cytometry analysis across the three titanium groups showed an increase in the proportion of cells expressing M2 markers, with Ti-MR and Ti-HR exhibiting a substantial rise compared to Ti-LR. These findings confirm the anti-inflammatory properties of Ti-MR and Ti-HR in comparison to the control and Ti-LR groups. Interestingly, a notable observation was made regarding M1/M2 ratio in the Ti-LR group: while the expression of M1-related genes decreased, the M1/M2 ratio of Ti-LR was the lowest among all groups, indicating a higher proportion of M2 macrophages compared to M1. This finding contrasts with the RT-PCR analysis, which revealed an increase in TNF-α gene expression, suggesting a heightened proinflammatory state. The discrepancy between the flow cytometry and RT-PCR results is likely because TNF-α is a nonexclusive cytokine from the macrophage perspective. It is known that subtypes of M2 macrophages, namely, M2b, M2c, and M2d, also produce TNF-α. 24 The microenvironmental effects of titanium are suspected to play a role in this phenomenon, where exposure to titanium may create conditions that transiently support proinflammatory cytokine gene expression while simultaneously promoting a transition to an anti-inflammatory (M2) phenotype over time. Thus, time-course analyses to map the dynamics of gene expression and phenotypic changes are essential for a more comprehensive understanding of these findings.

This study also identified nearly identical proportions of CD163 ^+^ and CD86 ^+^ cells in all groups, particularly Ti-HR, reflecting a high degree of macrophage plasticity and a wide range of immunophenotypes with overlapping characteristics. For instance, previous studies have reported that CD86 can be expressed by both M1 and M2 macrophages, despite their distinct functional profiles. 25 26 Similarly, CD163 is also expressed by all macrophages; however, its expression level in classically activated macrophages (M1) is significantly lower than in alternatively activated macrophages (M2). 27 Nevertheless, the overlapping cell populations observed in this study warrant further investigation in future research.

Our study presents evidence that macrophage-CM cultured with various titanium surface roughness enhances PDL MSCs proliferation. The Ti-MR surfaces consistently enhance MSCs proliferation at both 24 and 48 hours, suggesting that the CM from macrophages on Ti-MR surfaces positively impacts MSCs proliferation. This finding aligns with studies demonstrating that Ti-MR promotes M2 macrophages polarization, which is associated with the release of cytokines and growth factors that support MSCs proliferation and bone regeneration. 28 29 The consistent positive effect of Ti-MR surface may be due to the sustained release of supportive factors such as IL-10 and TGF-β from M2 macrophages, which facilitates MSCs growth and osteogenesis. 30 31 Overall, Ti-MR surfaces appear to provide consistently supportive environment for MSCs proliferation, underscoring their potential for enhancing regenerative outcomes in clinical applications. Further studies are needed to explore the underlying mechanisms and optimize titanium surface modifications for improved MSCs growth.

The regulatory role of RAW 264.7 macrophages in response to different samples on osteogenesis of MSCs were reported in this study and highlighted the significant impact of titanium surface properties and macrophage polarization on the osteogenic differentiation of PDL MSCs. These findings align with literatures indicating that M2 macrophages, particularly those promoted by Ti-MR surface, secrete cytokines such as IL-10 and TGF-β that facilitate osteogenesis. 32 33 The anti-inflammatory cytokines create a favorable microenvironment for MSCs differentiation into osteoblasts, enhancing bone formation. 34 35 36 37 In contrast, the role of M1 macrophages in osteogenic differentiation is more complex. The M1 macrophages are typically associated with proinflammatory responses, characterized by the secretion of cytokines like TNF-α and IL-1β, which can inhibit osteogenesis and contribute to a catabolic environment. 12 Although the study observed calcium nodule formation in the Ti-LR group despite of the upregulation of M1 markers, this could reflect an initial response to inflammation that may eventually inhibit bone formation due to sustained inflammatory environment. 38 39 40 Thus, while M1 macrophages might induce early response that supports MSCs activity, their long-term effects could be detrimental to osteogenesis if the inflammation persists.

The limitation of the present study includes its *in vitro* design, which demonstrates the potential to enhance the viability of Bone marrow-derived macrophages (BMDMs) and the osteogenic potential of PDL MSCs. However, the mineralization tendency of PDL MSCs should be assessed using a preclinical animal model in future studies. Despite the limitations, this study may provide a better understanding of surface characteristic-induced M1/M2 macrophage and facilitate the surface design generating desired immunological response toward dental implants in clinical practice.

## Conclusion

Titanium implant surface topography influences macrophage polarization and osteogenic differentiation of PDL MSCs, with Ti-MR being the most effective in polarizing macrophages toward M2 and inducing optimal osteogenic responses from PDL MSCs.

## References

[1] Zhu G Wang G Li J J Advances in implant surface modifications to improve osseointegration Mater Adv 2021 2 21 6901 6927

[2] Yan Y Wei Y Yang R Enhanced osteogenic differentiation of bone mesenchymal stem cells on magnesium-incorporated titania nanotube arrays Colloids Surf B Biointerfaces 2019 179 309 316 30981066 10.1016/j.colsurfb.2019.04.013

[3] Shin Y C Pang K M Han D W Enhanced osteogenic differentiation of human mesenchymal stem cells on Ti surfaces with electrochemical nanopattern formation Mater Sci Eng C 2019 99 1174 1181 10.1016/j.msec.2019.02.039 30889651

[4] Trindade R Albrektsson T Tengvall P Wennerberg A Foreign body reaction to biomaterials: on mechanisms for buildup and breakdown of osseointegration Clin Implant Dent Relat Res 2016 18 01 192 203 25257971 10.1111/cid.12274

[5] Hotchkiss K M Sowers K T Olivares-Navarrete R Novel in vitro comparative model of osteogenic and inflammatory cell response to dental implants Dent Mater 2019 35 01 176 184 30509481 10.1016/j.dental.2018.11.011

[6] Chen Z Bachhuka A Han S Tuning chemistry and topography of nanoengineered surfaces to manipulate immune response for bone regeneration applications ACS Nano 2017 11 05 4494 4506 28414902 10.1021/acsnano.6b07808

[7] Genin M Clement F Fattaccioli A Raes M Michiels C M1 and M2 macrophages derived from THP-1 cells differentially modulate the response of cancer cells to etoposide BMC Cancer 2015 15 01 577 26253167 10.1186/s12885-015-1546-9 PMC4545815

[8] Zhao T Chu Z Ma J Ouyang L Immunomodulation effect of biomaterials on bone formation J Funct Biomater 2022 13 03 103 35893471 10.3390/jfb13030103 PMC9394331

[9] Pitchai M Ipe D Tadakamadla S Hamlet S Titanium implant surface effects on adherent macrophage phenotype: a systematic review Materials (Basel) 2022 15 20 7314 36295379 10.3390/ma15207314 PMC9609829

[10] Satyanarayana ChP Raju L S Dumpala R Sunil B R A review on strategies to enhance the performance of the titanium based medical implants Mater Today Commun 2024 38 107985

[11] Li W Liu Q Shi J Xu X Xu J The role of TNF-α in the fate regulation and functional reprogramming of mesenchymal stem cells in an inflammatory microenvironment Front Immunol 2023 14 1.074863E6 10.3389/fimmu.2023.1074863 PMC9940754 36814921

[12] Mo Q Zhang W Zhu A Backman L J Chen J Regulation of osteogenic differentiation by the pro-inflammatory cytokines IL-1β and TNF-α: current conclusions and controversies Hum Cell 2022 35 04 957 971 35522425 10.1007/s13577-022-00711-7

[13] Orvalho J M Fernandes J CH Moraes Castilho R Fernandes G VO The macrophage's role on bone remodeling and osteogenesis: a systematic review Clin Rev Bone Miner Metab 2023 21(1–4):1 13

[14] Liang W Ding P Qian J Li G Lu E Zhao Z Polarized M2 macrophages induced by mechanical stretching modulate bone regeneration of the craniofacial suture for midfacial hypoplasia treatment Cell Tissue Res 2021 386 03 585 603 34568957 10.1007/s00441-021-03533-5

[15] Gao Q Rhee C Maruyama M The effects of macrophage phenotype on osteogenic differentiation of MSCs in the presence of polyethylene particles Biomedicines 2021 9 05 499 34062822 10.3390/biomedicines9050499 PMC8147332

[16] Zha K Tian Y Panayi A C Mi B Liu G Recent advances in enhancement strategies for osteogenic differentiation of mesenchymal stem cells in bone tissue engineering Front Cell Dev Biol 2022 10 824812 35281084 10.3389/fcell.2022.824812 PMC8904963

[17] Cai S Wu C Yang W Liang W Yu H Liu L Recent advance in surface modification for regulating cell adhesion and behaviors Nanotechnol Rev 2020 9 01 971 989

[18] Majhy B Priyadarshini P Sen A K Effect of surface energy and roughness on cell adhesion and growth - facile surface modification for enhanced cell culture RSC Adv 2021 11 25 15467 15476 35424027 10.1039/d1ra02402g PMC8698786

[19] Li J Wang X Lin Y Deng X Li M Nan C In vitro cell proliferation and mechanical behaviors observed in porous zirconia ceramics Materials (Basel) 2016 9 04 218 28773341 10.3390/ma9040218 PMC5502669

[20] Zhang Y Cheng X Jansen J A Yang F van den Beucken J JJP Titanium surfaces characteristics modulate macrophage polarization Mater Sci Eng C 2019 95 143 151 10.1016/j.msec.2018.10.065 30573235

[21] Veiseh O Vegas A J Domesticating the foreign body response: recent advances and applications Adv Drug Deliv Rev 2019 144 148 161 31491445 10.1016/j.addr.2019.08.010 PMC6774350

[22] O'Brien E M Spiller K L Pro-inflammatory polarization primes macrophages to transition into a distinct M2-like phenotype in response to IL-4 J Leukoc Biol 2022 111 05 989 1000 34643290 10.1002/JLB.3A0520-338R PMC9272532

[23] Hotchkiss K M Ayad N B Hyzy S L Boyan B D Olivares-Navarrete R Dental implant surface chemistry and energy alter macrophage activation in vitro Clin Oral Implants Res 2017 28 04 414 423 27006244 10.1111/clr.12814

[24] Strizova Z Benesova I Bartolini R M1/M2 macrophages and their overlaps - myth or reality? Clin Sci (Lond) 2023 137 15 1067 1093 37530555 10.1042/CS20220531 PMC10407193

[25] Olmes G Büttner-Herold M Ferrazzi F Distel L Amann K Daniel C CD163+ M2c-like macrophages predominate in renal biopsies from patients with lupus nephritis Arthritis Res Ther 2016 18 01 90 27091114 10.1186/s13075-016-0989-y PMC4835936

[26] Orecchioni M Ghosheh Y Pramod A B Ley K Macrophage polarization: different gene signatures in M1(LPS+) vs. classically and M2(LPS-) vs. alternatively activated macrophages Front Immunol 2019 10 1084 31178859 10.3389/fimmu.2019.01084 PMC6543837

[27] Itoh F Komohara Y Takaishi K Possible involvement of signal transducer and activator of transcription-3 in cell-cell interactions of peritoneal macrophages and endometrial stromal cells in human endometriosis Fertil Steril 2013 99 06 1705 1713 23461823 10.1016/j.fertnstert.2013.01.133

[28] Li Q Shen A Wang Z Enhanced osteogenic differentiation of BMSCs and M2-phenotype polarization of macrophages on a titanium surface modified with graphene oxide for potential implant applications RSC Adv 2020 10 28 16537 16550 35498860 10.1039/c9ra10563h PMC9052948

[29] Chen L Yao Z Zhang S Biomaterial-induced macrophage polarization for bone regeneration Chin Chem Lett 2023 34 06 107925

[30] Wang C Li T Zeng X Sustained delivery of IL-10 by self-assembling peptide hydrogel to reprogram macrophages and promote diabetic alveolar bone defect healing Dent Mater 2023 39 04 418 429 36931990 10.1016/j.dental.2023.03.014

[31] Mahon O R Browe D C Gonzalez-Fernandez T Nano-particle mediated M2 macrophage polarization enhances bone formation and MSC osteogenesis in an IL-10 dependent manner Biomaterials 2020 239 119833 32062479 10.1016/j.biomaterials.2020.119833

[32] Wang J Qian S Liu X M2 macrophages contribute to osteogenesis and angiogenesis on nanotubular TiO _2_ surfaces J Mater Chem B 2017 5 18 3364 3376 32264402 10.1039/c6tb03364d

[33] Chen X Wan Z Yang L Exosomes derived from reparative M2-like macrophages prevent bone loss in murine periodontitis models via IL-10 mRNA J Nanobiotechnology 2022 20 01 110 35248085 10.1186/s12951-022-01314-y PMC8898524

[34] Wei E Hu M Wu L TGF-β signaling regulates differentiation of MSCs in bone metabolism: disputes among viewpoints Stem Cell Res Ther 2024 15 01 156 38816830 10.1186/s13287-024-03761-w PMC11140988

[35] Kushioka J Chow S KH Toya M Bone regeneration in inflammation with aging and cell-based immunomodulatory therapy Inflamm Regen 2023 43 01 29 37231450 10.1186/s41232-023-00279-1 PMC10210469

[36] Putri A Pramanik F Azhari A Micro computed tomography and immunohistochemistry analysis of dental implant osseointegration in animal experimental model: a scoping review Eur J Dent 2023 17 03 623 628 36977479 10.1055/s-0042-1757468 PMC10569876

[37] Wang W Liu H Liu T Yang H He F Insights into the role of macrophage polarization in the pathogenesis of osteoporosis Oxid Med Cell Longev 2022 2022 2.485959E6 10.1155/2022/2485959 PMC9192196 35707276

[38] Torres H M Arnold K M Oviedo M Westendorf J J Weaver S R Inflammatory processes affecting bone health and repair Curr Osteoporos Rep 2023 21 06 842 853 37759135 10.1007/s11914-023-00824-4 PMC10842967

[39] Sun Z Yan K Liu S Semaphorin 3A promotes the osteogenic differentiation of rat bone marrow-derived mesenchymal stem cells in inflammatory environments by suppressing the Wnt/β-catenin signaling pathway J Mol Histol 2021 52 06 1245 1255 33566267 10.1007/s10735-020-09941-1

[40] Maruyama M Rhee C Utsunomiya T Modulation of the inflammatory response and bone healing Front Endocrinol (Lausanne) 2020 11 386 32655495 10.3389/fendo.2020.00386 PMC7325942

